# Cytokine and Chemokine Signals of T-Cell Exclusion in Tumors

**DOI:** 10.3389/fimmu.2020.594609

**Published:** 2020-12-14

**Authors:** Yu Zhang, Xin-yuan Guan, Peng Jiang

**Affiliations:** ^1^ Cancer Data Science Lab, Center for Cancer Research, National Cancer Institute, National Institutes of Health, Bethesda, MD, United States; ^2^ Department of Clinical Oncology, University of Hong Kong, Hong Kong, Hong Kong

**Keywords:** T-cell exclusion, cell therapy, immunotherapy, cytokine, chemokine

## Abstract

The success of cancer immunotherapy in solid tumors depends on a sufficient distribution of effector T cells into malignant lesions. However, immune-cold tumors utilize many T-cell exclusion mechanisms to resist immunotherapy. T cells have to go through three steps to fight against tumors: trafficking to the tumor core, surviving and expanding, and maintaining the memory phenotype for long-lasting responses. Cytokines and chemokines play critical roles in modulating the recruitment of T cells and the overall cellular compositions of the tumor microenvironment. Manipulating the cytokine or chemokine environment has brought success in preclinical models and early-stage clinical trials. However, depending on the immune context, the same cytokine or chemokine signals may exhibit either antitumor or protumor activities and induce unwanted side effects. Therefore, a comprehensive understanding of the cytokine and chemokine signals is the premise of overcoming T-cell exclusion for effective and innovative anti-cancer therapies.

## Introduction

Cancer immunotherapy aims to treat malignancies by leveraging the human immune system’s potential, especially cytotoxic T cells that target tumor-specific antigens. However, T-cell exclusion in tumors presents a significant factor for the adverse outcome of cancer patients and the resistance to cancer immunotherapies (1, 2). CD8 T cells and subgroups of CD4 helper T cells are primary contributors to antitumor immunity (3). Activated T cells need to penetrate the tumor core for their cytotoxic activity. Meanwhile, the infiltrating T cells need to survive, proliferate, and keep active in the hostile tumor microenvironment (TME). Cold tumors, characterized by a lack of effector T cells, can exclude T cells through many mechanisms, such as lack of tumor antigens, defect in antigen presentation, absence of T-cell activation, and the deficit of trafficking signals toward the tumor core (4).

Cytokines are small soluble proteins released by the malignant, stromal, and immune cells in the TME. Upon binding to their cognate receptors and triggering the intracellular pathway, cytokines can regulate the growth, apoptosis, activation, and differentiation of target cells (5). Chemokines, a subcategory of cytokines, provide the chemotactic signals for immune cell trafficking to specific destinations (6). Studies have reported significant correlations between the concentration of cytokines and chemokines with the prognosis of cancer patients (7–9).

This review will discuss different cytokines and chemokines that influence effector T-cell exclusion, focusing on T-cell trafficking, survival, and differentiation. We will also summarize cytokine-related therapies to promote T-cell infiltration and enhance antitumor responses. Elucidating the signaling mechanisms in T-cell exclusion and cytokine-mediated strategies to enhance the abundance of effector T cells at tumor sites is of great importance to the development of cancer immunotherapies.

## T-Cell Trafficking: T Cells Need to Penetrate the Battlefield

**Figure 1:**
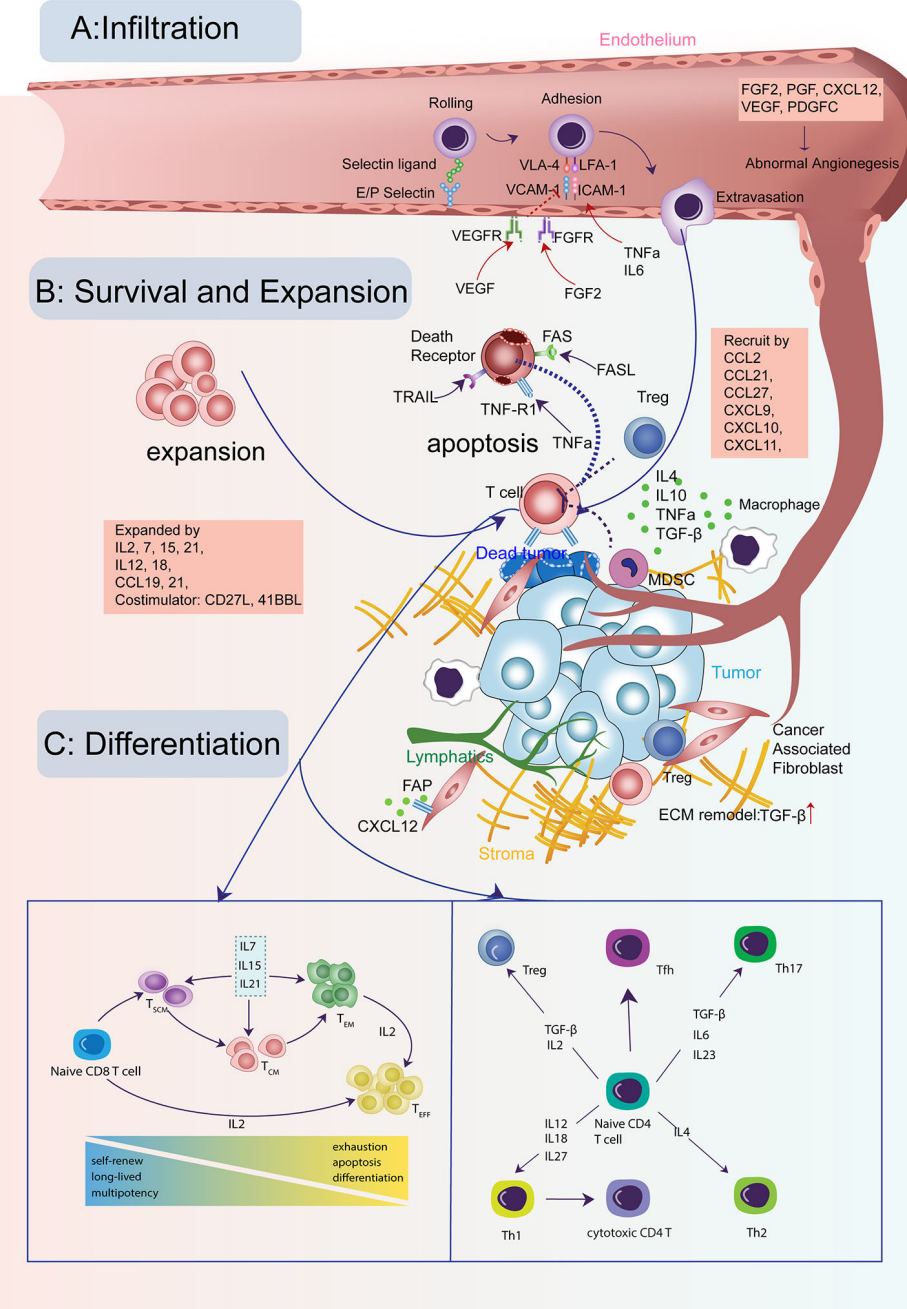
Cytokine signals in the life-span of T cells in tumors. **(A)** T cells need to infiltrate into the tumor for their cytotoxic activity. This process is guided through a set of cytokine, chemokine, and growth factor signals for T cells crossing the tumor vasculature and trafficking to tumor cores. **(B)** The infiltrating T cells need to survive and expand for sufficient numbers with the help from many cytokines and avoid the apoptosis induced by other signals. **(C)** Both CD8 and CD4 T cells need to keep active with the right differentiation path for its long-lasting tumor-fighting capability, modulated by many cytokines and growth factors.

A crucial factor for T-cell antitumor activity is the capabilities of specific and efficient trafficking. Upon primed and activated by antigen-presenting cells such as macrophages and dendritic cells (DC) in tumor-draining lymph nodes, T cells will migrate to tumor sites, exerting antigen-specific cytolytic functions. T-cell trafficking is a dynamic process involving rolling, tethering on the vascular endothelium (10), adhesion, extravasation, and chemotaxis (11) (**Figure 1A**).

### Signals for T Cells Crossing the Tumor Vasculature

The activated T cells will first gain the expression of homing molecules, including ligands for E- and P-selectin, which enable the rolling of T cells on the tumor vessel endothelium (12). Activated T cells also gain the expression of leukocyte adhesion protein LFA-1 (ITGAL) and VLA-4 (ITGA4), which bind to the intercellular adhesion molecule 1 (ICAM1) and vascular cell adhesion molecule 1 (VCAM1) on endothelial cells (11). Then, the T cells may transmigrate through the tumor vessel in response to various environmental stimuli. Cytokines such as IL6 and TNFa can enhance the endothelium adhesion activity by promoting adhesion molecules’ expression on tumor vessels (13, 14) (**Table 1**). One study demonstrated that IL6 signaling activated by systemic thermal therapy in a mouse model with the ovalbumin-expressing B16 tumor significantly upregulated E/P-selectin and ICAM1, which enhanced the CD8+ T-cell trafficking (13).

**Table 1:** Positive signals of T-cell infiltration.

Signal/Receptor	Producer/Target	Mechanisms	Therapy
IFNG/IFNGR1+IFNGR2	Cytotoxic T cells/endothelial cell, fibroblasts, tumor cell, monocyte	Interferon-γ induces CXCR3 ligands (CXCL9,10,11), thus enhancing the CXCR3-mediated T-cell recruitment (15).	Intravesical instillation of recombinant interferon-γ inhibits the recurrence of bladder tumors in patients (16).
(fn. 1) IL6/IL6R+IL6ST	T cell, macrophage, endothelial cell, epithelial cell/endothelial cell	IL6 trans-signaling enhances both E- and P-selectin interactions and ICAM1 dependent T-cell transmigration on tumor vessels (17).	IL6-rich tumor microenvironment provided by systemic thermal therapy improves cytotoxic T cells’ delivery to tumor lesions in mouse tumors and patient tumor explants (13).
IL12A+IL12B/IL12RB1 + IL12RB2	Phagocytic macrophage, DC/MDSC	IL12 may attenuate the impaired T-cell trafficking mediated by MDSCs by decreasing the percentage of MDSCs in tumors (18).	Treatment of IL12 significantly altered the phenotype and suppressive function of MDSC in mice (18).
(fn. 2) TNF/ITGAV	Lymphoid, mast, endothelial, fibroblast, tumor cell/ endothelial cell (19).	TNF stimulation induces ICAM1 and VCAM1 expression on endothelial cells for T-cell extravasation (14).	The systemic administration of TNF had severe toxicity (20). The fusion of TNF and ITGAV ligand had antitumor effects in mice (21)
CXCL9,10,11/CXCR3	Endothelial, fibroblast, tumor cell, monocyte/CXCR3+ T cell	These chemokines are induced by interferon-γ and share a receptor CXCR3, directing the migration of activated T and NK cells (22).	Virus-directed expression of CXCL10, 11, or recombinant CXCL10 injection in tumors can recruit anticancer T cells in mice (23–26).
CXCL16/CXCR6	Tumor cell, macrophage, DC, Fibroblast/activated T cell, Th1, NK cell	CXCL16 is chemotactic for cells expressing its receptor CXCR6 (27, 28).	
CCL2/CCR2	Endothelial cells/Th1, CD8 T	Actin fibers beneath the endothelial plasma membranes will dock vesicles storing CCL2. The tight lymphocyte-endothelial synapses will release the chemokine to promote the transendothelial T-cell migration (29).	CAR/CCR2 T cells displayed greater homing and tumor-killing in malignant pleural mesothelioma (30) and neuroblastoma tumors (31) in mice.
CCL3, 4, 5 (fn. 3)/CCR5	Epithelial cell, fibroblast, monocyte, NK cell, DC, endothelial cell, macrophage, lymphocyte/CCR5+ DC	CCL3,4,5 can indirectly promote effector T-cell recruitment by recruiting the DCs into tumor sites. Then, DC, in turn, recruits cytotoxic T cells into the tumor by producing CXCR3 ligands CXCL9 and CXCL10 (32, 33).	Combining adenoviral delivery of CCL3 with the adoptive transfer of activated effector T cells significantly attracted activated T cells to the murine melanoma tumors (34).
CCL21/CCR7	Lymphatic vessels, fibroblasts, HEV in lymph nodes/CCR7+ T cell, DC (35, 36)	CCL21 can significantly increase the CD4+ and CD8+ T cells, and DCs infiltration to tumors (37, 38).	Intratumoral injection of CCL21 induced DC and T-cell infiltration, causing tumor reduction in a murine lung cancer model (39).
CCL27/CCR10	Keratinocytes/CCR10+ skin-homing T cells	Skin-associated chemokine CCL27 is specifically expressed in epidermal keratinocytes and mediates the recruitment of skin-homing CCR10+ memory T cells to cutaneous sites (40).	The tumor injection of adenoviral vector encoding CCL27 attracted T cells and suppressed tumor growth in a murine melanoma model (41).

Besides the positive signals discussed above, there are negative signals that block effector T-cell adherence to the tumor endothelium and prevent the T cell recruitment to tumor sites (**Table 2**). The phenomenon is termed as endothelial cell anergy. For example, the angiogenic factors vascular endothelial growth factor (VEGF) and fibroblast growth factor 2 (FGF2) can cause endothelial cell anergy by repressing the expression of adhesion molecules ICAM1 and VCAM1 on the endothelium (76). Meanwhile, high levels of growth factors, such as VEGF, PDGFC (74), and PGF (75), can induce immature tumor angiogenesis. The aberrant vascular permeability and irregular blood flow that come with tumor vessels will cause inefficient effector T cells extravasation.

**Table 2:** Negative signals of T-cell infiltration.

Signal/Receptor	Producer/Target	Mechanisms	Therapy
IL35 (IL12A+IL27B)/(fn. 4) IL12RB2+IL6ST	Tregs, macrophages, B cell/T cell	Treg cells derived IL35 can decrease the intratumoral CD4+ and CD8+ T cells infiltration. Also, the infiltrated T cells displayed a less activated, effector memory phenotype (42).	Neutralization of IL35 enhances antitumor immunity in a genetically induced KP mouse model (42).
TGFB1/TGFBR1, 2, 3	Fibroblasts/Tumor epithelial cell	TGFβ is a well-known regulator of EMT. Fibroblast induced TGFβ may reprogram peritumoral stromal fibroblasts and exhibit a fibroblast- and collagen-rich tumor (43), which will decrease the CD8+ T effector cell penetration in the tumor (44).	Inhibitors of TGFβ and receptors have entered clinical trials (45–48). CAR-T cells expressing a dominant-negative TGF-βRII enhance T-cell expansion and prostate cancer eradication in clinical trials (49).
CXCL1, 2, 5/CXCR2	Tumor cell, macrophage, neutrophil/CXCR2+ MDSC	CXCL1, 2, 5- CXCR2 signal promotes the recruitment of MDSC to tumors (50).	Several CXCR2 antagonists and inhibitors have been tested in preclinical models and shown anticancer effects (51, 52).
(fn. 5) CXCL8/CXCR1, 2	Tumor cells, mast cells, TAM, endothelial cells/CXCR1, 2+ Neutrophil, MDSC	CXCL8 (IL8) enhances the infiltration of immune-suppressive cells expressing receptors (CXCR1, 2), such as tumor-associated neutrophils and MDSCs (53). T cells do not express CXCR1 and CXCR2 (54).	CXCR1 or CXCR2 modified CARs markedly enhance T-cell homing and persistence in murine GBM tumors (54).
CXCL12/CXCR4	FAP+ CAF/CXCR4+ MDSC, Endothelial cell, T cell	CXCL12 has chemo-repulsive effects on T cells (55). CXCL12 promotes angiogenesis by recruiting endothelial precursor cells (56). CXCL12 also recruits MDSCs to tumors (57).	AMD3100, a CXCR4 inhibitor, induced rapid T-cell accumulation around cancer cells in mice (58).
CCL2/CCR2	Endothelial cell, tumor cell, fibroblast, monocyte/CCR2+ TAM, MDSC, Treg	Soluble CCL2 promotes the recruitments of TAM, MDSC, and Treg to the tumor sites (59–61).	
CCL5/CCR5	Epithelial cell, fibroblast, monocyte, NK cell, DC, endothelial cell, macrophage, lymphocyte/CCR5+ TAM, Treg	CCL5 regulates TAM and MDSC migration (62). It can also recruit Treg to tumors (63).	
CCL17/CCR4, 8	DC, Endothelial cell/CCR4+, CCR8+ cells.	CCL17 induces chemotaxis in CCR4+ T cells, mainly Th2 and Tregs, generating an immunosuppressive TME (64, 65).	CAR- CD30 coexpressing CCR4 T cells have an improved homing and antitumor activity in the murine Hodgkin tumor model (66).
CCL22/CCR4	TAMs/CCR4+ Treg,	CCL22 enhances the recruitment of Tregs, thus decreasing effector T-cell homing (67).	
CCL28/CCR10	Tumor cells/CCR10+ skin-homing T cells	CCL28/CCR10 signals promote Treg recruitment in hypoxic tumors (68).	
VEGF/VEGFR1, 2, NRP1	Tumor, Macrophage, Endothelial, Fibroblast/Endothelial cell, Treg, MDSC	VEGF induces FASLG on endothelial cells, leading to T-cell apoptosis during extravasation (69). VEGF recruits the NRP1+ Tregs and VEGFR1,2+ MDSCs (70, 71).	The anti-VEGF/VEGFR is a standard therapy for many tumor types (72).
FGF2/FGFR1,2,3,4	CAF/Endothelial cells	FGF2 significantly blocks the adhesion molecules VCAM1 and E-selectin expression (73).	
PDGFC/PDGFRA	CAF/Endothelial cells	PDGFC acts as a proangiogenic signal (74).	
PGF/FLT1	TAMs/Endothelial cells	TAM production of PGF stimulates angiogenesis (75).	

These observations have suggested therapeutic approaches by targeting proangiogenic molecules. Anti-VEGF therapy produced synergistic antitumor responses with immune checkpoint blockade in phase I clinical trials of many cancer types (77). Also, anti-VEGF therapy resulted in significant clinical efficacy when combined with Chimeric Antigen Receptor (CAR)-T therapies by increasing tumor infiltration in humans (78). However, it is essential to note that the administration dose of anti-VEGF must be carefully considered. A low concentration of therapy could efficiently normalize tumor vasculature and enhance T-cell perfusion (79). However, a high dose of VEGF inhibitor will induce tumor-promoting hypoxic microenvironment by blocking the formation of tumor capillaries and causing insufficient oxygen supply (78, 80).

Notably, positive signals of T-cell extravasation may act as negative signals for T-cell recruitment in a context-specific manner. For example, tumor necrosis factor (TNF), which enhances T-cell adhesion molecules, may impair the effector T-cell infiltration by negatively regulating the formation of high endothelial venules (HEV) (81). HEVs are uniquely organized vessels in proximity to tumor sites and tightly associated with tertiary lymphoid structures (TLS). Tumors with HEV and TLS have a high density of effector T cells infiltration and favorable clinical outcome (82, 83).

### Chemotaxis for T Cells Trafficking to Tumor Core

After extravasation, T cells need guidance from environmental stimuli to arrive at tumor sites. This process is chemotaxis. The expression of chemokine CXC ligand (CXCL) 9, 10, and 11, secreted by tumor and stromal cells, are highly correlated to T-cell abundance in tumors of melanoma (84), lung (84, 85), and colorectal cancer (86). Moreover, high expression of CXCR3 ligands is significantly related to prolonged overall survival rate in cancer patients (87). Exploiting the therapeutic potential of CXCR3 ligands for antitumor T-cell recruitment has been successful in multiple preclinical models (23–26).

Notably, these CXCR3 ligands are all induced by interferon-γ, the effector cytokine secreted by cytotoxic T cells. However, intratumoral interferon-γ injection, which indeed increased the concentration of CXCL10 and CXCL11 in tumors, failed to induce the recruitment of effector T cells to human melanoma metastases in a clinical trial (88). Interferon-γ may upregulate the expression of PD-L1 and IDO1, leading to T-cell exclusion (89). A high level of Interferon-γ might compromise the combined anti-CTLA-4 and anti-PD-1 therapy in tumors with low tumor burden because Interferon-γ induced the apoptosis of activated antigen-specific T cells (90).

**Figure 2:**
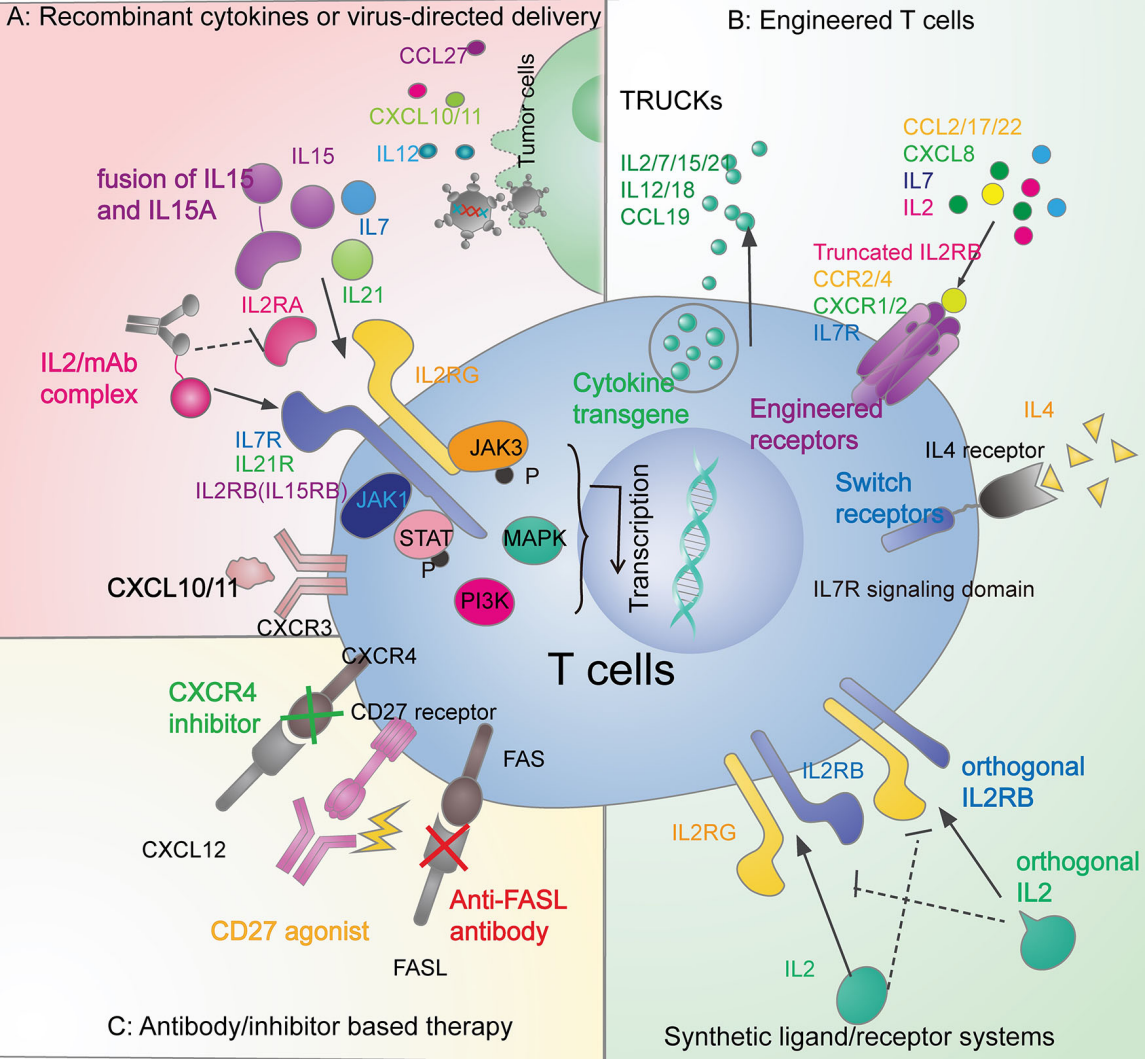
Therapeutic strategies in overcoming the T-cell exclusion by leveraging the cytokine signals. **(A)** Recombinant cytokines or virus-directed delivery. Administration of recombinant IL2 (94), IL7 (95), and IL15 (96, 97) have brought success in clinical trials. Also, there are other recombinant cytokines under research in preclinical models, such as IL21 (98), and CXCL10 (24). Adenoviral, retrovirus, and vaccinia vectors can also deliver cytokines, including CCL27 (41), IL12 (99, 100), CXCL10 (26), and CXCL11 (25). **(B)** Engineered T cells. The fourth-generation CAR T cells (TRUCKs) can release IL2 family cytokines (IL2, IL7, IL15, and IL21) (101), IL12, IL18 (102) and CCL19 (103). CAR T cells can also be engineered with functional receptors such as CCR2 (30, 31), CCR4 (66), CXCR1/2 (54), IL7R (54, 104), truncated IL2B domain (105), and switch receptors to overcome the immune-suppressive cytokines (106). Tumor-specific T cells can also be equipped with synthetic ligand/receptor systems such as IL2 and IL2RB orthogonal pairs (107). **(C)** Antibody/inhibitor-based therapy. Agonistic (105, 108) or antagonistic (109) antibodies and inhibitors (58) are applied to modulate cytokine signaling pathways in the anticancer immune response.

Besides CXCR3 ligands, other chemokines, such as CCL5, CXCL16, CCL21, and CCL27, may positively correlate with effector T-cell density within tumors (27, 37, 91). Recent studies showed that CCL5 may selectively mediate CD4 not CD8 effector T cells infiltration and enhance the therapeutic efficacy of checkpoint blockade in a mouse model of pancreatic cancer (92). However, the role of CCL5 on T-cell recruitment seems to be context-dependent. Another study showed that CCL5 upregulation is positively correlated with CD8 lymphocytes recruitment in the lung adenocarcinoma model (93). CCL27 is expressed in epidermal keratinocytes and mediates CCR10+T-cell homing to cutaneous tumors (40). One study employed an adenoviral vector to deliver chemokine CCL27 into tumors in a mouse model of B16BL6 melanoma and observed considerably increased recruitment of T cells (41) (**Figure 2A**).

### Negative Signals of T-Cell Trafficking Into Tumors

Although several chemokines are positive signals of T-cell infiltration as summarized above, the tumor microenvironment is a hostile environment containing many negative messages for effector T-cell chemotaxis. VEGF can inhibit NF-κB signaling induced endothelium activation, blocking the induction of chemokines CXCL10 and CXCL11, and impairing the T-cell infiltration (110). Another cytokine IL35, released by T regulatory cells (Treg), macrophages, or B cells (111), can limit antitumor T-cell recruitment and induce a significant decrease in the CD8: Treg ratio in a murine model.

Other factors reshaping the stromal structure will also contribute to reduced T-cell infiltration. Transforming growth factor-beta (TGFβ) is a crucial regulator of epithelial-mesenchymal transition (EMT) in tumor cells. TGFβ plays a critical role in T-cell exclusion since the abundant tumor-stromal compartments induced by EMT will trap effector T cells from penetrating the tumor core (112). One study from a cohort of metastatic urothelial cancer patients receiving anti-PD-L1 therapy found that lack of response was related to activation of TGFβ signaling in fibroblasts (112). Moreover, TGFβ secreted by stromal cells will also promote angiogenic factors like VEGF and recruit immune suppressive cells such as Treg and myeloid-derived suppressor cell (MDSC) in tumors, further creating an unfavorable environment for T-cell trafficking.

TGFβ blockade has demonstrated safety and efficacy in different stages of preclinical models and clinical trials (45, 46, 48). In a EMT6 mouse mammary carcinoma model that exhibited the T cell-exclusion phenotype, combining anti-TGFβ antibody with anti-PD-L1 therapy successfully blocked the TGFβ signaling in stromal cells, which facilitated T cells infiltration and tumor regression (112). Co-expression of a dominant-negative TGFβ receptor II of the CAR-T cells dramatically enhanced its potency of penetration, proliferation, cytokine secretion, and tumor-killing capability in prostate cancer mouse models (49). This therapy has entered Phase I clinical trial status. Additionally, M7824, a fusion compound targeting both TGFβ and PD-L1, is undergoing clinical testing as monotherapy and combination therapy with an adenovirus vaccine encoding the tumor-associated antigen TWIST1 (49, 113).

Many cytokines and chemokines have both well-defined functions and controversial roles in the literature, depending on their cellular context. For example, the tumor endothelium produces CCL2 stored in vesicles of actin fibers beneath the plasma membrane. The stored CCL2 may promote the transmigration capacity of T helper type 1 (Th1) and CD8 T cells after resting on the endothelium (29). However, the extracellular soluble CCL2, produced by variable cell types in tumors, may exert different functions in the antitumor immunity by recruiting macrophages, Tregs, and MDSCs (59–61). Although CCL2 has restricted clinical application, its receptor CCR2 could serve as a useful therapeutic target. CAR T cells engineered with CCR2 expression displayed satisfactory effector T-cell trafficking and antitumor efficacy in malignant pleural mesotheliomas (30) and neuroblastoma tumors (31) in mice.

Similarly to the application of CCR2, other cytokine-receptor pairs which contribute to immune-suppressive cells recruitment have shown therapeutic potentials in T-cell therapy. For example, CXCL8 recruits myeloid derived suppressor cells (MDSC) and neutrophils into tumors (53). CAR-T cells armed with CXCL8 receptors (CXCR1 and CXCR2) markedly enhanced T cells infiltration and led to complete antitumor responses in murine models ovarian cancer, pancreatic cancer, and glioblastoma (54). Another example is CCR4 receptor for CCL17 and CCL22, which recruit Treg and T helper type 2 (Th2) (64, 67). The CAR-CD30 T cells expressing CCR4 significantly enhanced the CAR T migration to tumor sites and achieved satisfactory tumor control in a Hodgkin tumor model (66).

## T-Cell Survival: T Cells Need to Survive on the Battlefield

### Signals Supporting T-Cell Survival and Expansion

Once T cells enter the tumor, the goal is to survive and proliferate in adequate numbers for efficient tumor killing (**Figure 1B**). Tumor infiltrating lymphocytes (TIL) manage to expand in the nutrient-deficient tumor microenvironment with the help of multiple cytokines (**Table 3**). Several studies have supported the pivotal role of the IL2 cytokine family (IL2, IL7, IL15, IL21) for CD8 T-cell expansion. IL2 was one of the earliest FDA-approved immunotherapies for metastatic melanoma and renal cell cancer (94, 117). Autologous TILs were transferred in conjunction with IL2 after *ex vivo* expansion with IL2, which mediated a durable and completed tumor regression in 22% of the heavily pretreated melanoma patients (143). However, reasons preventing IL2 from extensive clinical usage include short half-life *in vivo* (144) and severe toxicity at therapeutic dosage (145). Another barrier is that not only newly-activated CD8 T cells but also Tregs can express the trimeric high-affinity receptor IL-2Rαβγ (IL2RA+B+G complex) for IL2 signaling (116).

**Table 3:** Positive signals of T-cell survival.

Signal/Receptor	Producer/Target	Mechanisms	Therapy
(fn. 6) IL2/IL2RA,B,G	DC, activated T cells/effector T cell, Treg	CD8+ T cells depend on IL2 for sustained expansion (114, 115). However, the high-affinity IL2RA is not only expressed on activated T cells but also on Tregs, which is the primary barrier for the clinical application of IL2 (116).	IL-2 was one of the first FDA-approved immunotherapies for metastatic melanoma and renal cell cancer (94, 117), (fn. 7). Since IL2 expands effector T cells at the cost of Treg proliferation, engineered IL2 is necessary to preferentially target IL2 receptors on effector T cells (107).
IL7/IL7R+IL2RG	Fibroblastic reticular cell/T cell	IL7 promotes the homeostasis and expansion of naive and memory T cells by up-regulating BCL2 (118, 119), and suppressing pro-apoptotic mediators (120).	Coexpressing the IL7R with CAR-GD2 T cells activates STAT5 signaling and shows super antitumor response in metastatic neuroblastoma and glioblastoma mice model (104). Clinical trials using recombinant IL7 as monotherapy showed increases in CD4 and CD8 T cells with a decrease in Tregs in multiple cancer types (95, 121).
(fn. 8) IL10/IL10RA+IL10RB	Treg, Th1, DC, macrophage, epithelial cell/Tumor-resident T-cell (122).	IL10 directly activates and expands tumor-resident T cells without *de novo* infiltration from secondary lymphoid organs (123).	Treatment with pegylated IL10 restores tumor-specific CD8 T-cell accumulation and controls tumor growth in mice (124).
IL12A, IL12B/IL12RB1 + IL12RB2	Phagocytic cells, B cells, DC/T cells	IL12 stimulates activated T-cell proliferation (125).	Intratumoral injection of a recombinant retrovirus vector expressing IL-12 induces antitumor and anti-angiogenic effects in murine models of head and neck squamous cell carcinoma (99) and melanoma (100). The phase I trial of (fn. 9) NHS-IL12 in metastatic or locally advanced solid epithelial or mesenchymal tumors showed enhanced antitumor activity with increasing immune cell infiltration (126).
(fn. 6) IL15/IL15RA, IL2RB, IL2RG	Monocytes, macrophages, DC/CD8+ T cell	The IL15-IL15RA signaling triggers the downstream JAK1, JAK3, STAT3, and STAT5, which stimulates T-cell proliferation and survival (127). IL15 inhibits AICD and maintains T cells’ homeostatic proliferation.	IL15 has entered the clinical trial in patients with metastatic melanoma, renal cell carcinoma, and non-small cell lung cancer in CAR-T therapy and combination treatment with anti-PD1 antibody (96, 97, 128).
IL18/IL18R1+IL18RAP	Activated macrophages and Kupffer cells/T cells,	Combined stimulation with IL12 and IL18 can stimulate memory T cells in an antigen non-specific manner (129, 130). IL12 and IL18 may synergize with each other to induce Th1 differentiation (131).	
IL21/IL21RA, IL2RG	Th17, Tfh, NKT cells/T cell	IL21 synergistically works with IL15 to expand CD8+ memory T cells (98). IL21 also suppresses Foxp3-expressing cells (132).	The combined administration of IL21 and IL15 dramatically increased the CD8+ T cells and resulted in tumor regression in mice melanoma models (98). Likewise, the combination treatment of IL21 with IL7 promotes the expansion of CAR-T cells with a TSCM phenotype (133).
CD27L/CD27	T cells/T cells	The CD27L/CD27 signaling plays an essential role in T-cell differentiation, survival, and memory T-cell formation (19, 134).	CD27 agonist showed antitumor efficacy in mice models (135) and phase I and II clinical trials of advanced solid tumors (108, 136),
41BBL/41BB	DC, macrophages, B cells, T cells/activated T cell	41BBL facilitates cell activation, survival, and proliferation upon binding to 41BB on T cells (137).	41BBL armored CAR T-cells showed enhanced *in-vitro* and *in-vivo* efficacy (138).
CCL19/CCR7	Fibroblastic reticular cell/T cell	T zone fibroblastic reticular cells can prevent the death of naive and memory T cells by secreting CCL19 (139, 140).	CCL19 CAR-T achieved superior antitumor activity compared to conventional CAR-T in mice (103).
CCL21/CCR7, CXCR3	Lymphatic vessels, stroma cells, HEV in lymph nodes/CCR7+, CXCR3+ cells (35, 36)	CCL21 promotes the expansion of naive T cells in tumors (141).	Delivery of CCL21 to metastasis tumors enhances the ACT efficacy by promoting the T-cell survival and cytotoxic activity in mice (142).

Several methods may attenuate IL2’s propensity to promote Tregs expansion (146). Besides binding to IL-2Rαβγ, IL2 can stimulate naive and memory T cells expressing an intermediate affinity receptor IL-2Rβγ (IL2RB+G complex). Complexing IL2 with specific anti-IL2 antibodies will present IL2 to the intermediate (T effector) but not high-affinity (Treg) receptors, thereby reducing the Tregs production and causing massive CD8+ T cells expansion in mice (147–149) (**Figure 2A**). Engineered IL2 and IL2RB orthogonal pairs, consisting of the mutant IL2 cytokine and cognate mutant receptor in an engineered T cell, may transmit IL2 signals only to transferred T cells without interacting with the natural counterparts (106) (**Figure 2B**). Transduced CAR-T cells with a truncated IL-2Rβ domain increased STAT3 and STAT5 signaling and improved the CAR-T-cell expansion in mice leukemia and melanoma model (104) (**Figure 2B**).

Other members in the IL2 cytokine family play a similar and synergistic role with IL2 in cancer immunity through a shared gamma chain (CD132) in their receptor and downstream JAK-STAT signaling (133). For example, unlike IL2 produced by immune cells, IL7, mainly secreted by fibroblastic reticular cells in lymph nodes, can support the survival of naive and memory T cells expressing the receptor IL7R. IL7-IL7R signaling regulates the proliferation of target T cells *via* up-regulation of the anti-apoptotic BCL2 (118, 119) and suppression of pro-apoptotic mediators Bad and Bax (120). IL7 cannot trigger the expansion of Tregs because they express low levels of surface receptor IL7R. IL15 is essential for homeostasis and development of effector CD8 T cells and NK cells through interaction with its high-affinity receptor IL15RA. IL15 can also inhibit activation-induced cell death (AICD), further facilitating the proliferation of CD8 T cells (150). IL21, produced by T helper 17 (Th17), follicular helper T cells (Tfh), and NKT cells, drives NK expansion and differentiation (133). Also, IL21 inhibits Treg survival by downregulating the FOXP3 expression and favors the development of antigen-specific cytotoxic T cells (132).

In contrast to IL2, other IL2-family cytokines IL7, IL15, and IL21 do not induce Treg expansion. The role of these cytokines in modulating T cell-based cancer immunotherapies is currently being explored. Administration of recombinant IL7 alone showed a dose-dependent increase of T cells along with a decrease of Tregs in patients with lymphopenia (95). In clinical trials, human IL15 as monotherapy promoted proliferation of circulating NK cells and CD8 T cells in patients with metastatic melanoma and renal cancer, with the cost of severe toxicity at the therapeutic dose (96). Combined administration of IL15 and IL21 had synergistically accelerated the growth of both naive and memory CD8 T cells and resulted in tumor regression in a murine model of melanoma. IL21 has also demonstrated cooperative effects with IL7, but not IL2 (98).

Treatment of IL2-family cytokines in combination with TILs, CAR-T, and checkpoint blockade can lead to a broader and stronger antitumor response. Fourth-generation CAR-T cells armed with inducible cytokines have been defined as T cells redirected for universal cytokine-mediated killing (TRUCK) (**Figure 2B**). TRUCKs, loaded with IL2 family cytokines, enhance the persistence of CAR-T cells for antigen-specific tumor killing, and activate innate immune cells for antigen-negative tumors killing (101).

Besides the IL2 family, IL12 and IL18 secretion by TRUCKs have also been tested (151, 152). IL12 and IL18 can promote the activation and expansion of memory CD8 T cells in an antigen-independent manner (101). TRUCKs with inducible IL12 have dual antitumor activity. Firstly, the activated TRUCKs will lyse tumor cells and secret IL12 upon CAR engagement. Then, the locally released IL12 will not only promote CAR-T cells activation in an autocrine manner but also activate and recruit the innate immune cells (such as NK cells and macrophages) to kill tumor cells without antigens (102). Intratumoral administration of IL12 had antitumor effects in murine models of head and neck carcinoma (99) and melanoma (100). There are advantages of inducible production of IL12 (triggered by CAR signaling) over constitutive IL12 delivery at the tumor sites. As long as the TRUCKs are engaged and stay activated, there will be induction and secretion of IL12 for durable tumor control. It is important to note that the safety of TRUCKs is a concern since cytokine release syndrome happens in most CAR-T therapy (153).

Several cytokines, such as CD27L and 41BBL, which belongs to the tumor necrosis factor family, also act as costimulatory signals for T cells. CD27L is only transiently expressed on activated immune cells. However, the CD27L-CD27 costimulation bolsters T-cell activation, proliferation, and differentiation to a memory T-cell phenotype, thus enhancing anticancer immunity (134, 154). Targeting the CD27L-CD27 axis might be a therapeutic strategy given the observation that patients with CD27L or CD27 mutations and deletions are at a higher risk of developing Hodgkin lymphoma (155). Varlilumab, an anti-CD27 monoclonal agonistic antibody, showed clinical efficacy in a phase I study of refractory solid tumors (109). A combination of Varlilumab with checkpoint blockade therapy also established early success in phase I and II clinical trials (136).

The 41BBL-41BB signal’s importance is underscored by the observations that agonistic antibodies against 41BB can significantly promote CD8 T cells expansion and decrease T cells apoptosis, contributing to a robust antitumor immunity in mice (157, 158). However, the low efficiency (Utomilumab) (157) and liver toxicity (Urelumab) (158) severely hampered the clinical application of 41BB antibodies. The most remarkable clinical benefit of 41BB so far comes from the FDA-approved CAR-T cells containing 41BB as the intracellular costimulatory domain. The second-generation CAR-CD19 T cells armed with 41BBL have shown notable antitumor responses in several clinical trials against B-cell acute lymphoblastic leukemia (159, 160).

Interestingly, chemokines may sometimes enhance CD8 T cells’ survival and propagation apart from acting as chemoattractants. Both CCL19 and CCL21 can promote naive T cells’ survival (161). Secondary lymphoid organs are primary sources for CCL19 and CCL21, and access to the secondary lymphoid organs is crucial for naive T cells survival (139). Intratumoral delivery of CCL21 augmented the tumor-killing efficacy of adoptive cell therapy (ACT) in a murine model of melanoma by promoting T-cell survival rather than recruitment (142). IL10 can also induce the activation and multiplication of tumor-resident T cells without trafficking from the secondary lymphoid organs. IL10R, expressed on CD8 T cells, is necessary for the IL10-mediated tumor regression and the in-situ proliferation of CD8 T cells (123). However, given the suppressive function of IL10 during the T-cell priming and IL10-induced T cells exhaustion (162, 163), the value of IL10 as a therapeutic target needs further investigation.

### Signals Triggering T-Cell Apoptosis

There are various cytokine-mediated mechanisms by which the hostile TME triggers T-cell apoptosis (**Table 4**). The best-known one is through activation-induced cell death (AICD). AICD is a process that occurs when CD8 T cells express high FAS and FAS ligand (FASLG) expression levels upon activation, triggering the apoptosis of neighboring CD8 T cells (176). The binding of FAS to FASLG will recruit the FAS-associated death domain (FADD) to the intracytoplasmic death domain (IDD) of the receptor and initiates the caspase 8 activation and the subsequent cascade caspases (177). IL1, IL6, and TNF can also promote FAS and FASLG expression (178, 179). Moreover, the tumor endothelium can release FASLG, leading to apoptosis of T cells when they are trying to transmigrate the tumor vessel (69). Also, tumor-derived VEGF, IL10, and PGE2 can all enhance the FASLG expression (69). Notably, the endothelium-dependent apoptosis hardly works for Tregs because of the activation of anti-apoptotic molecules like BCL2 and CFLAR in Tregs (69, 180). An ovarian cancer study confirmed that the endothelium secreting FASLG induces deficient CD8 T cells infiltration and a predominance of Tregs (69).

**Table 4:** Negative signals of T-cell survival.

Signal/Receptor	Producer/Target	Mechanisms	Therapy
TNF/TNFR1, TNFR2	Lymphoid, mast, macrophage, endothelial cell, fibroblast, tumor cell/Activated T cell	TNF can mediate mature T-cell receptor-induced apoptosis through the TNFR1 (81). Macrophages can induce CD8 T-cell apoptosis *via* the interaction between macrophage membrane-bound TNFa (mbTNF) and TNFR2 on T cells (164).	TNF or TNFR1 blockade synergizes with anti-PD-1 on anti-cancer immune responses against solid tumors in mice (165).
FASLG/FAS	Tumor endothelial cell, NK cell, T cell/Activated T cell.	FASLG is a death ligand for activated T cells. Activated CD8+ T cells are sensitive to FASLG killing (166).	Anti-FASLG antibody treatment before adoptive T cells transfer significantly enhances CD8+ T cells infiltration in mice (69, 167).
TRAIL/TNFRSF10B (DR5)	Tumor cell, Treg cells/CAR- T cell	TRAIL hardly triggers T-cell apoptosis because T cells either lack or express low levels of functional death receptors (DR4 and DR5) (168). Interestingly, CAR-T cells may express DR5 and are prone to TRAIL-mediated apoptosis (169).	In vivo combined blockade of Fas and TRAIL signaling significantly rescued the CAR-T cells in mice (170).
TGFB1/TGFBR1, 2	Leukocyte, fibroblast, tumor cell/T cell	TGF-β impairs the cell cycle progression of CD4+ and CD8+ T cells (171). TGF-β inhibits IL2-dependent T-cell proliferation by suppressing the expression of IL2 and its receptor (172, 173).	Refer to **Table 2**
CCL5/CCR5	Epithelial cell, fibroblast, monocyte, NK cell, DC, endothelial cell, macrophage, lymphocyte/CCR5+ T cell	Tumor cells stimulate TILs to secret CCL5, which activates an apoptotic pathway in TIL involving cytochrome c release into the cytosol and activation of caspase-3 and -9 (174). CCL5 could enhance the Tregs’ killing ability on CD8+ T cells through TGF-β signaling (175). Cancer cells might induce CD4+ T cells to secrete CCL5 and activate the Fas-mediated apoptosis in CD8+ cells (47).	

Given the immune-suppressive role of FAS-FASLG signaling, different approaches to inhibit FASLG have been tested. For example, treatment with FASLG-neutralizing antibody (**Figure 2C**) markedly reduced T-cell apoptosis and cancer cell migration in a glioblastoma mouse model (109). Genetically engineered T cells with disruption of FAS-FASLG signaling introduced by adoptive T-cell transfer dramatically prevented FASLG-mediated T-cell apoptosis and achieved superior persistence in murine models (167).

Besides FASLG, other death receptors may mediate T-cell apoptosis when triggered by their cognate ligands, such as TNFRSF10A (TRAIL-R1, DR4), TNFRSF10B (TRAIL-R2, DR5), TNFRSF25, TNFRSF1A (TNFR1), and TNFRSF1B (TNFR2). Theoretically, the TNF-related apoptosis-inducing ligand (TRAIL) cannot induce apoptosis of T cells since T cells express low levels of TRAIL-R1/2, which contains the cytoplasmic death domain. TRAIL-R1/2 may recruit FADD, activates caspase 8, and leads to T-cell apoptosis upon binding to TRAIL (181). Interestingly, there was an observation that CAR-T cells did undergo programmed cell death triggered by the FAS-FASLG and TRAIL-DR5 pathway. Also, *ex vivo* combined blockade of FAS and TRAIL signaling significantly rescued the CAR-T cells (169, 170).

TNF receptors are also critical death receptors for activated T-cell apoptosis. Soluble and membrane-bound TNF bind to different receptors to trigger apoptosis. Upon activation, TNFR1 can either trigger cell apoptosis *via* the formation of FADD-IDD complex leading to caspase cascade activation or induce the proliferation pathway through NFkB activation. A study showed that T-cell depletion occurs in mice with FAS and FASLG defects. The inhibition of both FAS and TNF is necessary to eliminate T-cell death, and the TNF-TNFR1 signal mediates most CD8 T cells apoptosis (182). Macrophages can also induce apoptosis of CD8 T cells *via* the interaction between macrophage membrane-bound TNF and TNFR2 on T cells (164). Notably, MDSCs and Tregs also express TNFR2, whose activation will trigger the proliferation through NFkB signaling pathway instead of apoptosis in these cells (183).

Other mechanisms within the tumor microenvironment resulting in T-cell apoptosis include depletion of tryptophan by indoleamine-2,3-dioxygenase (IDO) and generation of Galectin 9, both of which binds to TIM3, which are predominantly mediated by immune-suppressive cells such as Tregs and MDSCs. Unexpectedly, chemokines can sometimes trigger non-classic apoptosis signals in T cells. TILs-secreted CCL5 induces CCR5+ (CCL5 receptor) T cells death through the release of cytochrome c from mitochondria and activation of caspase-3 and 8 (174). CCL5, secreted by tumor-infiltrating CD4 T cells, also facilitates the FAS-FASLG mediated CD8 T-cell apoptosis in gastric cancer (47).

## T-Cell Differentiation: T Cells Manage to Keep in their Proper States for Durable Fighting

### CD8 T Differentiation in Tumors

T-cell differentiation pathway is one of the primary factors determining T cells’ prolonged tumor-killing activity. Upon exposure to the cognate tumor-antigens, activated CD8 T cells will differentiate from a naive state into effector T cells. There are disputes regarding the differentiation track of CD8 T cells. The de-differentiation model purports that naive T cells are directly programmed into short-lived terminal effector T cells, followed by de-differentiation into memory cells with increased longevity (184, 185). Weissman and colleagues suggested that naive T cells differentiate along a sequential lineage path into stem-cell memory cells (TSCM), central memory cells (TCM), effector memory cells (TEM), and the terminally differentiated effector cells (184) (**Figure 1C**).

As terminal differentiation proceeds, T cells lose their self-renewal ability, proliferative potential, and lifespan (184, 186). The adoptive transfer of TSCM enhanced antitumor responses compared with TCM and TEM subsets in a humanized mouse model of mesothelioma (187). Given the superior anticancer efficacy of memory CD8 T cells, people have developed various T-cell selection methods for ACT, such as epigenetic and genetic modification (186, 188), reprogramming of induced pluripotent stem cells (189), and cytokine treatment.

The IL2 family members exert their specific roles in CD8 T-cell differentiation and proliferation (**Table 5**). IL2 drives terminal effector T cells differentiation and proliferation by upregulating perforin, granzyme B and IFN-γ and suppressing the memory cell marker BCL6 and IL7RA (199). On the contrary, IL7, 1L21, and IL15 may promote the memory cell phenotype. IL7 can generate TSCMs from their naive precursors (200, 201). CAR-T therapies frequently require IL7 during the ex-vivo expansion phase (202). For example, CAR-CD19 T cells cultured *in vitro* with IL7 and IL15 significantly induced a TSCM phenotype and produced a robust response against B-cell malignancies in phase I clinical trial (203). TRUCKs with constitutive IL7R signaling increased T-cell proliferation, survival, and tumor-killing activity upon exposure to tumor antigens, without stimulating bystander lymphocytes in murine cancer models (103).

**Table 5:** Positive signals of T-cell effector activity through differentiation.

Signal/Receptor	Producer/Target	Mechanisms	Therapy
IL7/IL7R+IL2RG	Fibroblastic reticular cell/CD8 T cell	IL-7 generated the stem-cell memory T cells from naive CD8 T cells (150).	
IL12A,B/IL12R IFN-α/β/IFNAR1, 2	Phagocytic cells, B cells, DC/CD8 T cells	IL12 and IFN-α/β provide a third signal, along with Antigen and costimulation, to support CD8 T memory programming, which involves chromatin remodeling and regulation of genes such as *TBX21* (*T-bet*) and *EOMES* (190, 191). IL12 also polarizes naive T cells into Th1 cells (192).	
IL15/IL15RA, IL2RB, IL2RG	Monocytes, macrophages, DC/CD8+ T cells	IL15 induces the generation of antigen-specific memory T cells (193).	
IL21/IL21R+IL2RG	Th2, NKT cells/CD8 T cell	IL21 suppresses the antigen-induced CD8+ T-cell differentiation from naive T cells to effector T cells and induced stem-like properties, which allows CD8+ T cells for secondary expansion after adoptive transfer (194).	
CD40L/CD40	DC, macrophages, B cells, T cells, mast cells/CD40+ T cell	CD40L/CD40 increases T-cell proliferation, CD8+ T-cell immunity, and memory (195).	
TGFB+IL6+IL23	In-vitro culture treatment.	CD4+ T-cell differentiates into Th17 *via* TGFB and IL6 (196). IL23 maintains the proliferation of Th17. (Fn. 10) Th17 are long-lived cells with stem-like properties. It can also convert into a Th1-lineage over time, switching from IL17 secreting cells to IFNγ producers or IFNγ/IL17A double producers (197).	Transfer of Th17 cells enhances survival and tumor regression better than Th1 cells in a murine melanoma model (198).
CCL21/CCR7, CXCR3	Lymphatic vessels, stroma cells, HEV in lymph nodes/CD4 T cells (35, 36)	CCL21 promotes Th1 polarization (141).	

Antigen-presenting cells secret IL15 bound together with its high-affinity receptor IL15RA (204). The signal will reach target cells that express IL-2Rβγ, including CD8 memory T cells. ALT-803, a fusion complex of IL15 and IL15RA receptor, exhibited a more substantial tumor-killing effect than native IL15 in preclinical models of myeloma through promoting the proliferation of CD8 memory T cells and inducing large amounts of IFN-γ (205). Administration of ALT-803 combined with anti-PD1 antibody showed tumor-killing effects in non-small cell lung carcinoma patients who failed the anti-PD1 monotherapy (97).

IL21 augments ACT therapy by preserving T cells in a younger phenotype but at the cost of less expansion than those expanded with IL2. However, adoptive T cells stimulated with IL21 induced a more robust antitumor response in a murine melanoma model (206). Similarly, IL21 treatment has generated TRUCKs with a more naive phenotype. When transferred into the host, these TRUCKs showed a dramatic propagation upon tumor exposure and achieved improved tumor control (194).

A study compared the therapeutic efficacy of CD19-specific TRUCKs equipped with IL2, 7, 15, and 21 expression cassettes in a murine lymphoma model. The result claimed that IL7 and IL21 were superior to IL2 and IL15 in enhancing tumor eradication, although IL2 and IL15 established increased effector functions. Interestingly, IL21 overexpression best supported the long-term persistence of memory T cells, while IL7-transduced T cells expanded to the greatest extent upon secondary antigen presentation (207). The varying roles of IL2 family members suggest that combinatorial approaches are necessary for ideal T cells-based therapy.

Other cytokines also contribute to CD8 T cell memory programming (**Table 5**). For example, the generation of memory cells needs signals from CD4 T helper cells. The interaction between CD40 expression on CD8 T cells and CD40 ligand (CD40L) expression on CD4 T cells is indispensable for the helper process. However, the CD40L/CD40 signal is not necessary for naive CD8 T cells to differentiate into terminal effector cells (195). IL12 and type I IFN (IFN-α/β) provide a third signal in concert with antigen presentation and costimulation to establish long-term memory CD8 T cells. IL12 and IFN-α/β promoted the memory program of naive CD8 T cells mainly through chromatin remodeling, which involved histone acetylation of genes like EOMES, TBX21, and GRZB (190). In addition, CD4 T helper cells provide a CD40-CD40L stimulus for DCs to produce IL12, promoting the CD8 T cell memory program (190, 209).

### Functional Fate of CD4 T-Cell Subsets in Tumors

Unlike CD8 T cells, the differentiation of CD4 T cells is divergent. CD4 T cells differentiate into various T helper (Th) cell and regulatory (Treg) cell lineages to exert their functions in the tumor immunity. Upon exposure to different lineage-determining cytokines during activation, naive CD4 T cells may have several distinct effector fates, such as Th1, Th2, Th17, Treg, and Tfh (**Table 5** and **Figure 1C**). Besides supporting CD8 T cells, CD4 T cells may directly lyse tumor cells in an MHC-II dependent manner through the secretion of perforin and granzyme (209, 210). A recent study demonstrated the occurrence of clonal expanded cytotoxic CD4+ T cells by single cell sequencing, these CD4 T cells possessed lytic capabilities against autologous tumors (210). Multiple studies have identified several origins of CD4 cytotoxic T cells, such as Th2, Th17 and Treg (211). However, the majority of CD4 CTL are thought to come from IFN-γ secreting Th1 cells (212).

Each of the CD4 T subsets has a specific role in antitumor immunity by producing and receiving distinct cytokines. Th1 can provide IFN-γ, TNF, CCL2 and CCL3 to enhance the recruitment of CD8 T cells, NK cells and anticancer macrophages (213–216). Cytokines IFN-γ, IL12, IL18, IL27 can promote Th1 polarization, thus contributing to tumor control (141, 192). However, the role of Th2 cells in the tumor immunity is contradictory and context-dependent. IL4 is both the inducer of Th2 polarization (**Table 6**) and the effector cytokines secreted by Th2. IL4 has an immune-suppressive role by antagonizing the Th1 response and supporting Tregs (219). CAR-T cells engineered to overcome the immune-suppressive nature of IL4 have brought early success. CAR-MUC1 T cells with an inverted IL4 receptor exo-domain plus an IL7 receptor signal endo-domain (**Figure 2B**) facilitated a potent antitumor response in a mouse model of breast cancer with an abundance of IL4 in the tumor milieu (106). Th2 may also promote angiogenesis and hinder apoptosis of tumor cells by remodeling the cytokine environment for macrophages and eosinophils infiltration (220). However, some studies have demonstrated the antitumor activity of Th2 cells by recruiting the innate cells such as eosinophils to the tumor (221).

**Table 6:** Negative signals of T-cell effector activity through differentiation.

Signal/Receptor	Producer/Target	Mechanisms	Therapy
(fn. 11) IL4/IL4R+IL2RG	Th2, basophils, eosinophils, mast cells, NKT cells, Adipose tissue, cancer cells/Th1	(fn. 12) IL4 drives CD4+ T-cell polarization into the Th2 phenotype and suppresses IFNγ-producing Th1 cells (217).	MUC1 CAR T with a cytokine switch receptor of IL4 receptor extracellular domain fused to an IL7 intracellular signaling domain can proliferate and suppress tumor growth in mice breast cancer model (106).
TGFB1/TGFBR1,2,3	Leukocyte, fibroblast, tumor cell/CD4 T cell	TGFβ promotes the conversion of effector T cells to Tregs (218).	

The signals of TGFβ and IL10 polarize naive CD4 T cells to Tregs (**Table 6**). Tregs have been regarded as exerting suppression roles in antitumor immunity and high Treg/CD8 ratio in tumor infiltrates correlates with poor prognosis in cancer patients (222). However, recent study showed that IFN-γ production by Tregs is necessary for the therapeutic responses of anti-PD1 in a mice model, which shed light on characterizing the contribution of IFN-γ+ Tregs in tumor immunotherapy (223).

Naive CD4 T cells can also differentiate into Th17 cells with stimulation by TGFβ and IL6 and maintenance with IL23. Characterized by the high production of IL17 and IL22, Th17 cells have a controversial role in the cancer immunity context. Th17 cells may stimulate angiogenesis and promote tumorigenesis. However, these cells also serve as tumor-suppressive cells by stimulating effector CD8+ T cells, supporting immune cell recruitment, and transitioning to Th1-lineage over time (224). Moreover, Th17 cells possess long-lived memory-like properties through the expression of stem-cell markers such as CCR7, LEF1, and TCF7 (225). Thus, the adoptive transfer of Th17 polarized CAR-T cells had been shown to produce superior tumor regression than Th1 cells in mice (225, 226). Follicular helper (Tfh) T cells characterized by IL21 secretion have been shown to contribute to antitumor responses by promoting the formation of intratumor follicular structures, which were positively associated with prognosis of cancer patients (227, 228).

Since the cytokine environment is pivotal in fate-determination of naive CD4 T cells, manipulation of CD4 T cells using cytokine signals has provided substantial promise in therapy. For example, Th17 polarized cells in cell culture with TGFβ, IL6, and anti-IFN-γ antibody (preventing Th1 differentiation) mediated effective tumor eradication and a survival advantage in a murine melanoma model (198). However, the lifetime of CD4 T cells generated is very short, and many other cytokines are necessary for their maintenance, which represents a significant barrier for use in clinical applications (229). Robust methods are still lacking for generating effector CD4 T subtypes with stem-like properties and longevity.

## Cytokine Release Syndrome: Every Coin Has Two Sides

As reviewed in previous sections, many therapies modulate cytokine and chemokine signaling to overcome the T-cell exclusion barriers in tumors (**Figure 2**). However, a danger of cytokine-based treatments is the severe toxicity from cytokine release syndrome (CRS). CRS is the over-activation of the immune system characterized by a flood of inflammatory cytokines, fever, and multiple organ dysfunction (230). CRS can happen after administration of therapeutic monoclonal antibodies or cytotoxic chemotherapies, such as the CD28 agonist TGN1412 (231), Rituximab and Obinutuzumab (targeting CD20) (232, 233), Dacetuzumab (targeting CD40) (234), Nivolumab (anti-PD1) (235), Oxaliplatin (236), and Lenalidomide (237).

Moreover, T-cell therapies, such as CAR-T and TCR-T cells, the bispecific T-cell (BiTE) single-chain antibody, and the dual-affinity re-targeting antibody, have produced the high CRS frequencies (238). CRS happens in nearly all CAR-T clinical trials, with presentations ranging from mild symptoms such as fever to life-threatening manifestations, including sepsis, thromboembolism, neurotoxicity, and multi-organ failure (230, 239). However, there is no conclusive evidence connecting the CRS severity to the immunotherapy response, and complete tumor remission can happen in patients without CRS (240).

The exposure of CAR-T cells to a tumor antigen can trigger CRS. The activation and proliferation of CAR-T cells release primary cytokines such as IL1, IFN-γ, and TNF, which induce the activation of other immune cells, such as macrophages, DCs, and monocytes (241). These cells then produce excessive amounts of secondary cytokines, such as IL6, IL10, and IL5 (242). Among these cytokines, IL6 is a crucial regulator of CRS and contributes to critical symptoms (243). The IL6/IL6R complex binds to the membrane-bound IL6ST (gp130) and activates a cascade of intracellular signaling, which results in severe CRS (244).

Several clinical factors are potentially predictive of the CRS severity. The first factor is the tumor burden. In many observations, the most severe CRS only occurs after the first administered dose, and will not occur during the subsequent therapies, called the first-dose effect. It is believed that the first-dose effect is due to the high tumor antigen load at the initiation of treatment (245). The administered dose of an agent is another factor (246). The maturation of the immune system may be another factor for CRS because children are more likely to develop severe CRS following CD19 CAR-T-cell infusion in clinical trials. Also, the type of T-cell engaging agents affects the onset, duration, and severity of CRS (160). For example, first-generation of CAR T cells hardly triggered the CRS because of the lack of a costimulatory domain. Among the second-generation CAR-T cells (247), CARs with CD28 costimulation have a higher CRS rate than those containing a 41BB co-stimulation (248). Before CAR-T infusion, the lymphocyte depletion type also affected the risk, with a higher CRS incidence observed after fludarabine-based lymphodepletion (249).

The principle of CRS management is to prevent life-threatening toxicity and preserve the maximum antitumor immune responses. Low-grade CRS can be treated symptomatically with antipyretics and fluid therapy. As for severe CRS, in the BiTE blinatumomab context, some clinical trials advised the usage of corticosteroids to reduce the CRS incidence (250). Because clinicians can give BiTE repeatedly, immediate action can be taken upon CRS manifestation, even at the cost of lowering the antitumor response. However, unlike BiTE, the manufacture of CAR T cells allows only limited amounts for one-time administration. Therefore, corticosteroids should be avoided at the first-time treatment of CRS in patients receiving CAR T therapies, unless severe neurotoxicity has developed.

IL6 levels are significantly higher in the serum of patients with severe CRS after CAR-T treatment. IL6 and IL6 receptors are attractive targets for CRS treatment because IL6 is less critical for T-cell function than other inflammatory cytokines. The FDA approved Tocilizumab (monoclonal antibodies against IL6R) to treat severe CRS in patients who are at least two years old (251). For patients who are unresponsive to anti-IL6 or IL6R treatment, other ongoing clinical trials are evaluating T cell-depleting antibodies, such as Alemtuzumab, IL-1R-based inhibitors (Anakinra), and Ibrutinib (252, 253).

## Conclusions and Future Directions

The systematic categorization of cellular signaling mechanisms that modulate the T-cell infiltration, survival, and differentiation in tumors is the premise of overcoming the T-cell exclusion barriers. However, many cytokines have both positive and negative effects on the anticancer immune response depending on their different receptor usage, cellular context, and interactions with other signals. Meanwhile, cytokines promoting the T-cell functions may also induce the life-threatening cytokine release syndrome without clear indicators. Given a large number of signaling molecules and the intricate crosstalks among them, it remains a significant challenge for the field to gain a comprehensive view of the complicated immune ecosystem.

Leading-edge technologies such as single-cell omics and spatial genomics have enabled the profiling of the tumor microenvironment at high dimensions. Many of these new technologies, coupled with computational models, can reveal the cytokine and chemokine activities through the molecular status of their downstream signaling pathways. Meanwhile, the rapid development of automated technologies has enabled the large-scale screening of immunological assays in preclinical models. Catalyzed by recent technological advances, we foresee rapid knowledge growth on cytokines and chemokines.

Only two cytokines interferon-alpha and IL2 have been approved by the FDA for treatment of refractory melanoma and renal cell cancer, and are rarely used as monotherapy. Bottlenecks in the therapeutic application of cytokine therapies include the dose-limiting toxicity, the short half-life in the circulation, the low concentration at tumor sites when administered intravenously, and the unwanted recruitment of immune suppressive cells. Advancements in delivery technologies have shown encouraging safety and efficacy in preclinical models. For example, smart nanocarriers can respond to multiple stimuli in the blood circulation and tumor by changing their physical and chemical properties for precise and lasting cytokine release (254, 255).

T cells armed with stimulatory cytokines have great potential to remodel the suppressive tumor microenvironment. However, there remains a need to generate “smarter” T cells, given the multiple suppressive signals in tumors, the inter-and intra-tumor heterogeneity, and potential severe toxicities of current T cell-based therapies. Modern gene-editing technologies can encode immunomodulatory fusion proteins in T cells to rewire the inhibitory or death receptor signaling. Such engineered T cells should sense the host environment and react to different cues in a precise manner. The synthetic Notch receptors could serve as a versatile platform for engineered T cells. Activation of Notch and the intracellular transcriptional signal upon the customized antigen sensing should trigger production of a specific cytokine profile (256).

Given the complexity of tumor heterogeneity, it is unlikely that one therapeutic solution is sufficient to overcome the T-cell exclusion barriers in tumors. We foresee that future successful treatments will leverage rational combinations among different therapy modules after elucidating specific cytokine activities in patient samples through high-resolution genomic technologies.

## Author's Note

1. IL6 has a dual function in the tumor microenvironment. It has a dark face that acts on tumor cells through multiple intrinsically downstream mediators to support cancer cell proliferation, survival, and metastatic dissemination. IL6 also works on other cells within the tumor stroma, promoting angiogenesis and tumor evasion (17).

2. TNF plays a dual role in cancer immunity, TNF induced T-cell adhesion is ICAM1 and VCAM1 dependent, and most of the chemo-attracted cells are Tregs (257), Bregs (258, 259) and MDSC (259), which are negative modulators of the immune response. TNF also triggers the activation-induced cell death of CD8 T lymphocytes (**Table 4**) and impairs tumor infiltration by CD8 T lymphocytes (**Table 2**).

3. CCL5 has a tumor-promoting role by inducing tumor cell proliferation (260), angiogenesis, and matrix metalloproteinases (47). It also suppresses the antitumor immune response by increasing the recruitment of TAM and Treg in tumors (63). Moreover, it stimulates the apoptosis of CD8 T cells (**Table 4**).

4. The IL35 receptor components vary by cell type. In T cells, IL-35 binds IL6ST (gp130) and IL12Rβ2. In B cells, IL35 signals through IL12Rβ2 and IL27RA (WSX-1) (5, 261).

5. CXCL8 has a tumor-supporting role by activating the epithelial-mesenchymal transition (262), promoting angiogenesis (262, 263), and stemness potential (264).

6. IL2 and IL15 share the β and γ components of their receptors and have similar functions on T cells, including stimulating proliferation of cytotoxic T lymphocytes. However, IL2 promotes terminal differentiation and elimination by AICD (265), but IL15 inhibits AICD and promotes the generation of long-lived stem memory T cells and maintains homeostatic proliferation (266). Other cytokines sharing the same γ-chain in their receptors (defined as the γc cytokine family) include IL4, IL7, IL9, and IL21 (150).

7. Reasons that prevent the extensive usage of IL2 for cancer therapy include short half-life *in vivo* (144), severe toxicity at therapeutic dosage (145), and propensity to promote Treg proliferation (146).

8. Treg-derived IL10 drives the exhaustion of CD8 T cells in tumors through the up-regulation of several inhibitory receptors (PD1, LAG3, and TIGIT) *via* a BLIMP1 dependent pathway (162). IL10 also inhibits the activation of CD8 T cells by decreasing antigen sensitivity (163).

9. NHS stands for the antibody NHS76 against DNA released by necrotic tumor cells.

10. Th17 is a double-edged sword in anticancer immunity. It may secrete high levels of the characteristic cytokine IL17 to stimulate angiogenesis and tumorigenesis. In contrast, Th17 may stimulate the effector CD8 T cells as a tumor-suppressive factor (224).

11. IL4 exerts controversial functions based on cancer types. In breast cancer, It promotes tumor growth by suppressing the effector function of Th1-polarized T cells (267). However, it drives the survival of B cell and T cells in other cancer types and promotes the long-term development of CD8 T memory cells (217).

12. Some studies demonstrated the antitumor activity of Th2 cells in collaboration with tumor-infiltrating granulocytes (268).

## Author Contributions

YZ and PJ outlined the review structure, finished the literature survey, and wrote the manuscript. X-yG participated in the discussion. All authors contributed to the article and approved the submitted version.

## Funding

The work is sponsored by the NIH intramural research program and Bau Tsu Zung Bau Kwan Yeu Hing Research and Clinical Fellowship from the University of Hong Kong.

## Conflict of Interest

The authors declare that the research was conducted in the absence of any commercial or financial relationships that could be construed as a potential conflict of interest.

## Abbreviations

ICB, immune checkpoint blockade; TME, tumor microenvironment; CRS, cytokine release syndrome; HEV, high endothelial venule; TLS, tertiary lymphoid structure; AICD, activation-induced cell death; EMT, epithelial-mesenchymal transition; ACT, adoptive cell therapy; BiTE, bispecific T-cell; TIL, tumor-infiltrating lymphocyte; DC, Dendritic cell; TSCM, T stem-cell memory cell; TCM, T central memory cell; TEM, T effector memory cell; Th1, T helper type 1; Th2, T helper type 2; Th17, T helper type 17; Tfh, follicular helper T cell; Treg, T regulatory lymphocyte; MDSC, myeloid-derived suppressor cell; TAM, tumor-associated macrophage; CAR, Chimeric Antigen Receptor; CAF, cancer-associated fibroblast.
